# Cure of Acute Mania by Pleuro-Pneumonia

**Published:** 1844-11-02

**Authors:** 


### CURE OF ACUTE MANIA BY PLEURO-PNEUMONIA.

Dr. Fabrege states in the "Journal de Medécine," that he has repeatedly seen insane patients recover their reason previous to death—a circumstance which he attributes, in common with other authors, to the revulsion which a severe disease exercises on the brain. In the following case this salutary revulsion was followed by a return to health:—

A man, 30 years of age, soldier in a regiment of the line, was placed in the Montpelier Asylum. He had served in Africa, where he contracted intermittent fever, which gave way to quinine, and had returned home on convalescent leave. It was whilst in his family that he manifested the first symptoms of insanity. These symptoms were, at the onset, looked upon as the result of intoxication, he not being a model of sobriety. On his admission he was labouring under acute intermitting mania. The mania gradually became continued. During the rigorous winter of 1837, he often escaped into the yard, in a state of complete nudity. At the beginning of April he was attacked with severe double pleuru-pneumonia, and immediately became perfectly reasonable. The pleuro-pueumonia gave way under the usual treatment, and he returned home on the 22d, perfectly rational.—*London Lancet.*

